# Turn left or turn right? Lessons from the management of intraoperative cardiac arrest due to Type I Kounis syndrome: a case report

**DOI:** 10.3389/fmed.2026.1766987

**Published:** 2026-04-30

**Authors:** Yuanyuan Rong, Zhaolei Tian, Tao Hu, Haishan Feng, Lei Wang, Yong-be Zhao, Jianfeng Fu, Aiyu Zhang, Huaqin Liu

**Affiliations:** 1 Department of Anesthesiology, The Fourth Hospital of Hebei Medical University, Shijiazhuang, China; 2 The Second Department of Thoracic Surgery, The Fourth Hospital of Hebei Medical University, Shijiazhuang, China; 3 Department of Cardiac Surgery, The Fourth Hospital of Hebei Medical University, Shijiazhuang, China

**Keywords:** allergy, anesthesia, case report, Kounis syndrome, thoracoscopic lobectomy

## Abstract

Kounis syndrome (KS), also known as allergic angina syndrome, is a potentially life-threatening perioperative condition that may be underrecognized under general anesthesia. Management is challenging because anaphylaxis and acute coronary events can occur concurrently and progress to cardiac arrest. We report a 70-year-old man undergoing elective left thoracoscopic lobectomy under general anesthesia. During the procedure, he developed abrupt hypotension and ST-segment elevation followed by ventricular fibrillation (VF). Cardiopulmonary resuscitation (CPR) was initiated, and 1 mg intravenous (IV) epinephrine was administered. The first defibrillation attempt failed to restore an organized rhythm. Marked erythema on both upper extremities was noted and intraoperative anaphylaxis was suspected. Despite anti-anaphylaxis treatment and two additional defibrillation attempts, there was no response. A cardiologist was consulted and internal defibrillation was initiated. After opening the pericardium, papaverine was administered. Sinus rhythm was obtained after the fourth defibrillation, with a total resuscitation time of 29 min. The patient was transferred to the intensive care unit (ICU) and was ultimately diagnosed with Type I Kounis syndrome. He was discharged without complications. This case suggests that in patients without underlying coronary artery disease, reversible coronary vasospasm may represent a pre-dominant pathophysiological mechanism in Type I Kounis syndrome. Early consideration of coronary vasodilator therapy, alongside standard anaphylaxis management, may therefore be warranted in selected perioperative settings.

## Background

KS is a potentially life-threatening perioperative entity that may be underrecognized, particularly under general anesthesia (1–3). Its management is challenging because anaphylaxis and acute coronary events can occur simultaneously and may progress to cardiac arrest requiring CPR (4). Because typical ischemic symptoms cannot be reported under anesthesia, recognition often relies on hemodynamic instability and dynamic ECG changes, which may delay diagnosis and treatment. We report a perioperative case of Type I KS under general anesthesia to highlight key diagnostic and management considerations.

## Case description

A 70-year-old man was electively admitted for left video-assisted thoracoscopic surgery in the right lateral decubitus position. He had no medical history of hypertension, diabetes, or coronary artery disease, and no known drug or food allergies.

At 2:40 p.m., baseline vital signs were as follows: non-invasive blood pressure (NIBP) 150/62 mmHg, SpO_2_ 97%, and heart rate (HR) 62 beats/min. General anesthesia was induced with remimazolam 15 mg, sufentanil 30 μg, and cisatracurium 12 mg. Tracheal intubation was performed uneventfully. Anesthesia was maintained with a continuous remifentanil infusion and sevoflurane 1.5%−2.0%. Shortly after induction, localized papules appeared on the right upper limb, accompanied by bradycardia (HR 43 beats/min) and hypotension (BP 90/52 mmHg). An intravenous bolus of ephedrine was administered, after which BP increased to 112/50 mmHg and HR to 68 beats/min.

At 4:22 p.m., the patient developed sudden ST-segment elevation with marked hypotension. A norepinephrine bolus followed by continuous infusion was administered. Telemetry showed resolution of the ST-segment changes as BP increased to 140/72 mmHg. At 4:30 p.m., VF occurred, followed by cardiac arrest. The inspired oxygen fraction was increased to 100%, and CPR was initiated. The initial defibrillation attempt was unsuccessful. Generalized rash was noted after the sterile drapes were removed, and intraoperative anaphylaxis was suspected; antihistamines and corticosteroids were administered immediately. Despite IV epinephrine, lidocaine, and amiodarone, subsequent defibrillation attempts at 4:40 p.m. were unsuccessful. At 4:48 p.m., an experienced cardiac surgeon was consulted, the pericardium was opened, and internal defibrillation was performed, but no organized rhythm was restored. At 4:55 p.m., papaverine 30 mg was administered intravenously, and an additional 30 mg (diluted in 10 ml normal saline) was applied topically to the cardiac surface. After the fourth defibrillation at 4:59 p.m., sinus rhythm was restored, although the HR was approximately 30 beats/min. Atropine 5 mg was administered intravenously, and the HR increased to 70–80 beats/min. Given the marked response, an additional 30 mg of papaverine in 500 ml of normal saline was administered as a continuous IV infusion. By 5:28 p.m., vital signs had stabilized. The operation was completed, and the patient was transferred to ICU at 6:00 p.m.

**Figure 1:**
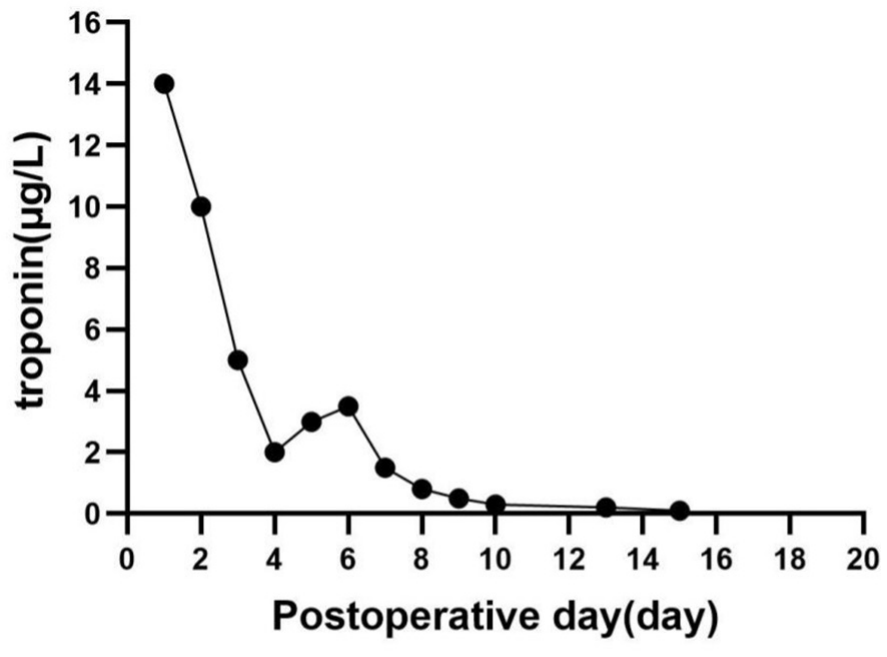
Post-operative changes in troponin levels. The normal reference range for troponin is 0–0.034 μg/L.

On post-operative day (POD) 4, coronary angiography revealed no significant stenosis in the major coronary arteries. The endotracheal tube was removed on POD 8, and the patient was able to ambulate independently on POD 15. Post-operative troponin levels over time are shown in Figure 1. The patient was discharged on POD 29 without complications (Table 1).

**Table 1:** Chronological timeline of perioperative events, physiologic changes, interventions, and clinical reasoning.

Time	Clinical findings	Monitoring changes	Interventions	Clinical reasoning
2:40 p.m.	Induction of anesthesia	NIBP 150/62 mmHg; HR 62 bpm	Remimazolam; sufentanil; cisatracurium	Baseline status
A few minutes after induction	Localized papules on the right upper limb	HR 43 bpm; BP 90/52 mmHg	IV ephedrine bolus	Initial consideration: allergic/anesthetic-related reaction
4:22 p.m.	Sudden ST-segment elevation	Marked hypotension	Norepinephrine bolus and infusion	Consideration of coronary involvement
4:30 p.m.	Ventricular fibrillation; cardiac arrest	No effective circulation on ECG monitoring	CPR; IV epinephrine 1 mg	Resuscitation according to guidelines
4:40 p.m.	Defibrillation unsuccessful	Persistent VF	Anti-allergic therapy; repeat defibrillation	Suspected allergy-related mechanism
4:48 p.m.	Pericardium opened; cardiac massage and internal defibrillation performed	No return of spontaneous circulation (ROSC)	Cardiac surgical team involved	Refractory resuscitation
4:55 p.m.	Ongoing cardiac massage and defibrillation	No ROSC	Papaverine (30 mg IV; 30 mg topical to the cardiac surface)	Coronary vasospasm strongly suspected
4:59 p.m.	Sinus rhythm restored after the 4 th defibrillation	HR ~30 bpm	Atropine 5 mg	Relief of vasospasm considered
~5:00 p.m.	Post-ROSC bradycardia	HR increased to 70–80 bpm	Continuous IV papaverine infusion (30 mg diluted in 500 ml normal saline)	Maintain coronary vasodilation
5:28 p.m.	Hemodynamics stabilized	Vital signs stable	Ongoing monitoring/supportive care	Stabilization achieved
6:00 p.m.	Post-operative transfer	Vital signs stable	Transferred to ICU after completion of surgery	Post-resuscitation management
POD 4	Coronary angiography	No significant stenosis	—	Type I Kounis syndrome confirmed
POD 8	Extubation	Stable	Endotracheal tube removed	Respiratory recovery
POD 15	Mobilization	Clinically stable	Ambulated independently	Functional recovery
POD 29	Discharge	No complications	Discharged without complications	Favorable outcome

## Discussion

KS refers to acute coronary events triggered by the massive release of inflammatory mediators during an allergic or hypersensitivity reaction. Based on the underlying coronary status, KS is generally classified into three variants: Type I occurs in patients with angiographically normal coronary arteries and is characterized pre-dominantly by reversible coronary vasospasm; Type II occurs in patients with pre-existing atherosclerotic disease and involves plaque disruption and/or thrombosis triggered by an allergic reaction; and Type III is associated with stent thrombosis (5–7). In our patient, post-operative coronary angiography revealed no significant stenosis or evidence of plaque rupture, which is more consistent with Type I Kounis syndrome.

The perioperative setting is a high-risk context for KS because multiple potential sensitizing exposures may occur within a short time frame. Common triggers include anesthetic-related agents (e.g., remimazolam, sufentanil, cisatracurium), perioperative antibiotics, and colloid solutions. These exposures may activate mast cells through IgE-dependent or non–IgE-dependent pathways and initiate an inflammatory cascade. In this case, localized papules and transient hypotension developed shortly after induction of anesthesia, suggesting that a hypersensitivity response may have started early. Because multiple potential allergens are often present perioperatively and no allergen-specific testing was performed, a single culprit cannot be identified, and a multifactorial trigger cannot be excluded.

Under general anesthesia, patients cannot report chest pain or other typical ischemic symptoms. Therefore, perioperative recognition of KS relies largely on hemodynamic instability and dynamic intraoperative electrocardiographic changes (5). In our patient, ST-segment elevation and malignant arrhythmias developed after the allergic manifestations appeared, suggesting coronary involvement. Prior reports have noted that some cases present initially only with hypotension or rash and are recognized later when ST changes emerge, leading to delayed diagnosis (2). KS has been reported more frequently in men aged 40–80 years, and symptoms typically occur shortly after exposure to the triggering agent (4, 8). Accordingly, when dynamic ECG changes or worsening hemodynamic instability occur in the setting of an allergic reaction, clinicians should maintain a high index of suspicion for coronary involvement.

From a differential diagnosis standpoint, the first step is to distinguish perioperative anaphylaxis from KS. Perioperative allergic reactions commonly present with generalized features such as rash, hypotension, and bronchospasm, and they often improve rapidly with anti-allergic treatment, without persistent evidence of myocardial ischemia. However, when dynamic ST-segment changes, malignant arrhythmias, or poor response to standard anti-allergic management accompany an allergic presentation, coronary involvement should be strongly considered. Other acute coronary entities should also be excluded. Primary acute coronary syndrome is an important consideration; although our patient developed ST elevation and VF, post-operative coronary angiography showed no significant stenosis or plaque rupture, and there was no history of atherosclerotic disease, arguing against a typical plaque-rupture myocardial infarction. Takotsubo syndrome can also present with stress-related ST changes and arrhythmias, but it is usually accompanied by characteristic apical ballooning and reversible left ventricular dysfunction, for which supportive imaging evidence was lacking in this case. Isolated coronary spasm may mimic these findings; however, in our patient, systemic allergic manifestations and hemodynamic fluctuations preceded the coronary event, and the response to the vasodilator papaverine was pronounced, making an inflammatory mediator–driven coronary smooth muscle spasm more plausible. Considering the temporal sequence, exclusion by imaging, and treatment response, the diagnosis in this case favors Type I Kounis syndrome. Key features distinguishing perioperative anaphylaxis from KS in anesthetized patients are summarized in Table 2.

**Table 2:** Key differential features between perioperative anaphylaxis and Kounis syndrome (under general anesthesia).

Domain	Anaphylaxis	Kounis syndrome
Core mechanism	Systemic hypersensitivity reaction with peripheral vasodilation and capillary leak	Allergic reaction with concomitant coronary involvement (vasospasm and/or plaque disruption)
Common perioperative triggers	Anesthetic agents, perioperative antibiotics, colloids, blood products	Similar perioperative triggers (often drug-related)
Typical onset	Usually within minutes after exposure	Also usually within minutes after exposure
Cutaneous manifestations	Common (rash, flushing)	Common (often present in Type I)
Hypotension	Common	Common
Bronchospasm	May occur	May occur
Dynamic ST-segment changes	Usually absent	Common (ST elevation or depression)
Malignant arrhythmias	Uncommon	May occur (VT, VF)
Cardiac biomarkers	Usually normal	May be elevated
Coronary angiography	Normal	Type I: normal; Type II: plaques and/or thrombosis may be present
Response to epinephrine	Usually effective	May be suboptimal and/or worsen ischemia
Response to vasodilators	No clear benefit	Often improves (nitrates, calcium channel blockers, papaverine)
Key perioperative clues under general anesthesia	Predominantly hypotension and/or bronchospasm	Allergic manifestations plus dynamic ECG changes or otherwise unexplained arrhythmias
Perioperative “red flags”	Hypotension with stable ECG	Allergic manifestations with ST changes or poor response to resuscitation
Key distinguishing point	No evidence of coronary ischemia	Evidence of coronary ischemia and/or vasospasm

Therapeutically, determining the priority between treating an allergic reaction and managing an acute coronary process is a major clinical challenge in the perioperative setting. Because KS often involves concurrent hypersensitivity and coronary involvement, clinicians must dynamically balance these two pathophysiologic processes. In our patient, ST-segment elevation rapidly progressed to VF, and CPR was initiated immediately in accordance with current resuscitation guidelines, including epinephrine administration (9). However, in suspected Type I KS, epinephrine may improve allergy-related circulatory compromise yet also increase myocardial oxygen demand and enhance coronary vasoconstriction, thereby precipitating or worsening myocardial ischemia and vasospasm (10). In this case, when conventional resuscitative measures had limited effect, return of spontaneous rhythm occurred after intravenous and topical papaverine, suggesting that reversible coronary vasospasm may have played a dominant role in the circulatory collapse, consistent with Shibuya et al.'s (11) report of spastic contraction of coronary smooth muscle during KS events. From a clinical perspective, sinus rhythm was restored following the administration of papaverine. This temporal association supports the hypothesis of coronary vasospasm to some extent; however, given the nature of a single-case observation, a definitive causal relationship cannot be established.

Accordingly, for life-threatening allergic reactions, epinephrine should remain a first-line agent as recommended by current guidelines (12). Nevertheless, when clinical clues suggest concomitant coronary vasospasm, the route and dose of epinephrine should be reassessed cautiously under close monitoring, and coronary vasodilator therapy should be initiated early in parallel. Nitrates or calcium channel blockers (CCB) may help relieve functional flow limitation, and papaverine can be considered as a vasodilator option. Papaverine can be considered as a vasodilator option due to its direct smooth muscle relaxation effect, which may help relieve coronary vasospasm in Type I Kounis syndrome. However, we acknowledge that systemic vasodilation induced by papaverine may potentially worsen hypotension, particularly in hemodynamically unstable patients. In our case, papaverine was administered under close hemodynamic monitoring, and the decision was based on a risk–benefit consideration, prioritizing the reversal of suspected coronary vasospasm after conventional resuscitative measures had failed. Meanwhile, antihistamines and corticosteroids may attenuate the inflammatory cascade and reduce the risk of sustained vasoconstriction. Based on these mechanistic considerations and our clinical observations, we drafted a perioperative management approach for suspected Type I KS (Table 3) to support clinical decision-making.

**Table 3:** Suggested perioperative management considerations for suspected Type I Kounis syndrome.

Item	Phase 1: allergy-dominant	Phase 2: overlap (allergy + coronary involvement)	Phase 3: vasospasm-dominant
Predominant pathophysiology	Mast cell activation; mediator surge	Mediator-driven coronary constriction; endothelial dysfunction	Severe reversible coronary vasospasm; functional flow limitation
Typical features	Rash; hypotension; bronchospasm; no persistent ST	Dynamic ST; hemodynamic instability; arrhythmia tendency	Persistent ST; VT/VF; poor response to resuscitation
Priority	Anaphylaxis: high Vasodilation: low	Anaphylaxis: mod–high Vasodilation: mod–high	Anaphylaxis: maintenance Vasodilation: high
Anaphylaxis management	Fluids; H1 antihistamine; H2 blocker; corticosteroid; epinephrine (per guideline)	Continue antihistamine + corticosteroid	Maintain basic anti-allergic therapy
Coronary vasodilation	Not prioritized	Nitrates; CCB (diltiazem/verapamil); nicorandil; papaverine	Prioritize nitrates; CCB; papaverine (IV/topical); nicorandil (if needed)
Epinephrine strategy	Standard per guideline	Cautious; balance α/β effects	Highly cautious; avoid excessive α-mediated vasoconstriction
Key focus	Control systemic allergy; stabilize circulation	Control allergy + improve coronary perfusion	Relieve vasospasm; restore flow; limit ongoing ischemia

Mechanistically, the central pathological basis of Type I KS is excessive mast cell activation (6). Following initiation of an allergic response, mast cell degranulation releases histamine, leukotrienes, platelet-activating factor, and multiple inflammatory cytokines. These mediators can directly provoke coronary smooth muscle contraction and may further amplify vasoconstriction by impairing endothelium-dependent vasodilation (13). Emerging evidence suggests a direct relationship between mast cell activation and endothelial dysfunction. In patients with systemic mastocytosis, serum tryptase levels have been shown to correlate inversely with flow-mediated dilation, indicating that greater mast cell activation is associated with poorer endothelium-dependent vasodilatory function (14). This finding provides biological plausibility for the combination of mediator-driven coronary vasospasm and endothelial dysfunction in Type I KS. In this context, coronary spasm is unlikely to reflect a purely reflexive smooth muscle response, but rather an interaction among inflammation, endothelium, and vascular smooth muscle.

Overall, in perioperative patients with suspected KS, management should shift from a single-disease framework to an integrated, mechanism-oriented strategy. Dynamic assessment of the pre-dominant pathophysiology and timely adjustment of treatment priorities are essential to enable individualized decision-making.

Importantly, this case differs from previously reported case series by providing a detailed perioperative and anesthesia-oriented perspective. In contrast to prior reports that primarily describe clinical outcomes, our case emphasizes real-time decision-making in a high-stakes intraoperative setting, where anaphylaxis and coronary ischemia must be addressed simultaneously. This highlights the complexity of competing priorities and offers practical insights for anesthesiologists managing similar scenarios.

## Conclusion

In summary, this case suggests that in patients without underlying coronary artery disease, reversible coronary vasospasm may represent a pre-dominant pathophysiological mechanism in Type I Kounis syndrome. Early consideration of coronary vasodilator therapy, alongside standard anaphylaxis management, may therefore be warranted in selected perioperative settings. This case also underscores the importance of an integrated, mechanism-based approach in perioperative critical events, rather than relying solely on standard single-pathway management strategies.

## Data Availability

The original contributions presented in the study are included in the article/supplementary material, further inquiries can be directed to the corresponding author.
